# Genomic evolution of ST228 SCCmec-I MRSA 10 years after a major nosocomial outbreak

**DOI:** 10.1128/jcm.00203-24

**Published:** 2024-06-27

**Authors:** Florian Mauffrey, Claire Bertelli, Gilbert Greub, Laurence Senn, Dominique S. Blanc

**Affiliations:** 1 Infection Prevention and Control Unit, Infectious Diseases Service, Lausanne University Hospital and University of Lausanne, Lausanne, Switzerland; 2 Institute of Microbiology, Lausanne University Hospital and University of Lausanne, Lausanne, Switzerland; Universität Münster, Münster, Germany

**Keywords:** MRSA, outbreak, genomics, THD, random forest

## Abstract

In this study, we investigated the genomic changes in a major methicillin-resistant *Staphylococcus aureus* (MRSA) clone following a significant outbreak at a hospital. Whole-genome sequencing of MRSA isolates was utilized to explore the genomic evolution of post-outbreak MRSA strains. The epidemicity of the clone declined over time, coinciding with the introduction of multimodal infection control measures. A genome-wide association study (GWAS) identified multiple genes significantly associated with either high or low epidemic success, indicating alterations in mobilome, virulence, and defense mechanisms. Random Forest models pinpointed a gene related to fibrinogen binding as the most influential predictor of epidemicity. The decline of the MRSA clone may be attributed to various factors, including the implementation of new infection control measures, single nucleotide polymorphisms accumulation, and the genetic drift of a given clone. This research underscores the complex dynamics of MRSA clones, emphasizing the multifactorial nature of their evolution. The decline in epidemicity seems linked to alterations in the clone’s genetic profile, with a probable shift towards decreased virulence and adaptation to long-term carriage. Understanding the genomic basis for the decline of epidemic clones is crucial to develop effective strategies for their surveillance and management, as well as to gain insights into the evolutionary dynamics of pathogen genomes.

## INTRODUCTION

*Staphylococcus aureus* is a versatile and ubiquitous bacterial pathogen that can cause a wide range of infections, from mild skin and soft tissue infections to severe and potentially fatal conditions, such as pneumonia, sepsis, and endocarditis (1). *S. aureus* is notorious for its ability to become resistant to penicillin derivatives mainly due to *mecA* genes and to other antibiotics, such as quinolones, gentamicin, and sulfamethoxazole/trimethoprim (2). Phylogenetic studies showed that infections caused by antibiotic-resistant strains often occur in epidemic waves initiated by successful clones. Methicillin-resistant *S. aureus* (MRSA) features prominently in these epidemics. The introduction of methicillin in 1960 marked the first wave of MRSA with the archaic clone ST250 (3). Subsequent waves in the 1970s and 1990s featured new strains, including community-associated MRSA with smaller SCCmec allotypes (IV and V) and the Panton Valentine leukocidin virulence factor (4). The epidemiology of MRSA has been described as highly dynamic, and clonal replacement of predominant clones at a given location was widely documented (5–7). However, the reasons why some clones replace others are still unclear.

Rapid genomic evolution is a characteristic defining many pathogenic bacteria, including *S. aureus*. The genetic variability of these pathogens enables them to adapt to changing host environments and to evade the immune system. This genomic plasticity significantly contributes to the emergence of new virulent strains and the acquisition of antibiotic resistance. Mobile Genetic Elements (MGEs) have a key role in accelerating and promoting the adaptation of *S. aureus* to environmental pressures (8, 9), by either acquisition or loss of genetic elements and/or by mutations modifying gene expression or regulation. For instance, the USA500 strain became hypervirulent after an IS element (IS256) was acquired by horizontal transfer and was inserted into the promoter sequence of a repressor of toxins (the protein Rot) (10). Another example is the success of the USA300 strain that seemed to be correlated with the acquisition of the *speG* gene and the Arginine Catabolic Mobile Element (ACME) (11). Nevertheless, what will make a lineage successful is largely unknown and still difficult to predict. Therefore, there is a need to track these changes to follow, study, and understand what determines the epidemiological success of this pathogen.

Traditionally, studies have focused on virulence traits acquisition that could explain the spread and virulence of pathogens in the population. However, no studies focused on the genomic changes occurring in an epidemic clone after a hospital outbreak. At the University Hospital of Lausanne, a molecular epidemiological surveillance of MRSA that was in place since the mid 1990s showed the emergence and decline of different clones (12–15). The last one (ST228-I) was of particular interest as it was responsible for two nosocomial outbreaks, one in 2001 and the second from 2008 to 2012, involving over 1,600 patients and representing 280–700 cases annually (16). The phylogeography of this clone was studied using whole genome sequencing and showed that it first emerged in South Germany and was introduced in 1999 in Switzerland (17).

Although it is possible to estimate the epidemic success of a lineage or clone by establishing its phylogeny and studying its epidemiology, it is difficult to associate drivers of epidemic success using this information. Time-scaled Haplotypic Density (THD) has been developed as a metric representing epidemic success, giving the possibility to highlight such drivers (18–20). It has been shown to be more accurate to evaluate epidemic success than traditional operational definitions based on epidemiological studies (21). So far, this metric has been essentially used to study *Mycobacterium tuberculosis* and the spread of antibiotic resistances (22–25). A recent work used THD to identify mutations characterizing the epidemiological success of strains across different clonal complexes but failed to find significant candidates (21).

In this study, we analyzed the genomes of various ST228-I isolates that were collected up to a decade after the end of the last outbreak and compared them with isolates from the original outbreak. We reconstructed the phylogeny of the ST228 clone at both international and local levels to investigate the clone evolution. We also examined changes in the clone’s accessory genome to identify any potential adaptations that may have occurred after the outbreak. Additionally, we attempted to identify genetic markers that could represent the post-outbreak evolution of the clone using THD as a metric of the epidemic success.

## MATERIALS AND METHODS

The University Hospital of Lausanne is a 1,100 bed tertiary care hospital where screening for MRSA of at-risk patients was performed for over three decades. At least one isolate per patient was typed by a molecular method (initially pulsed-field gel electrophoresis followed by double locus sequence typing) (26). From 2013 to 2018, up to 10 isolates from new ST228 MRSA cases were selected annually, for a total of 56 ST228 isolates. Whole genome sequencing of these 56 isolates was performed on an Illumina MiSeq platform (European Nucleotide Archive [ENA], accession project number PRJEB49254). These 56 isolates were compared with 365 ST228 isolates from a previous study (17) selected to represent the diversity of Swiss isolates from 1999 to 2012 (*N* = 250) and European isolates (*N* = 115) (Table S1). Bioinformatics analyses were performed in Bionumerics V8.1.1 (bioMérieux, Applied Maths NV, St Martens Latem, Belgium) with default parameters. Reads were mapped against the reference genome N315 (16), and SNPs were identified with the strict filtering option (close SNP set). A *de novo* assembly was performed using the Unicycler option in Bionumerics. Based on the *de novo* assemblies, acquired resistance traits, acquired virulence traits, resistance loci, virulence loci, resistance mutations, and pathogenicity islands were analyzed using the *S. aureus* functional genotyping plugin v2022.12.05 available in Bionumerics. These data sets were exported for further analyses. The SNP alignment was also exported and curated by removing all positions with gaps and missing data.

A maximum likelihood (ML) tree of the concatenated SNPs was generated using IQ-tree v2.2.0.3 with a transversion model with equal base frequency and ascertainment bias correction (TVMe+ASC). The best fitting evolutionary model was estimated with ModelFinder (27), and 500 bootstraps were used to build the final consensus tree. Tree visualization and annotation were carried in R v4.2.2 using the ggtree package v3.6.2. The final tree was rooted on the oldest German isolate (RKI 97–00028). Based on this tree, a subset of 218 isolates was created, comprising only isolates from the 2008–2012 Lausanne outbreak clone and their descendants for subsequent analyses. In this data set, each isolate corresponded to a different patient. A ML tree using this data set of 218 isolates was generated with the same parameters and rooted on the identified index case (isolate 18412).

THD was calculated as a proxy for strain epidemic success from SNP data (18) using the the package v1.0.1 (https://github.com/rasigadelab/thd) in R with the following parameters: m = 2814816 (effective genome size for *S. aureus*), mu = 1.87e^−6^ (ST228 mutation rate), (17) and t = 1. A time scale of 1 year was chosen to model rapid and short-term events that correlated with ST228 clone dynamic. To classify isolates as having either high or low epidemic success, a regression model was used to fit THD values using a polynomial equation. The high/low epidemic success threshold was determined by identifying the inflection point in the model, representing the point where THD exhibited a decrease towards the end of the outbreak. All isolates with THD values eceeding this threshold were categorized as high epidemic success (HES), whereas those below the threshold were classified as low epidemic success (LES). Based on this classification and the outbreak pylogenetic tree, three LES clusters were defined. Correlation between the acquired resistance traits and epidemic success was tested using Fisher’s exact test. The acquired virulence traits data sets showed no variation among these isolates and were not tested.

All subsequent analyses were performed using default parameters if not specified. Assembled genomes were annotated using Bakta v1.6.0. Bakta annotation files were used to conduct a pan-genome analysis using Roary v3.13.0, gathering all putative proteins into orthologous groups. A genome-wide association study (GWAS) was conducted using Scoary v1.6.1 with the Roary presence/absence table as input (28). Genes inherited together were concatenated (--collapse), and 1,000 permutations were used to check if significance was not reached by chance. The phylogenetic tree of the Lausanne outbreak was provided to Scoary, rather than using the internally calculated tree, to account for the population structure effect. Four different comparisons were made: each LES cluster against all HES isolates, and all LES isolates against all HES isolates. Genes with an adjusted *P* < 0.05 (Benjamini–Hochberg), and an empirical *P* < 0.05 were considered significantly associated with epidemic success. COG categories were manually attributed to all these genes if not present in the annotations, when possible.

A Principal Coordinates Analysis (PCoA) based on the accessory genome profiles was performed using Jaccard distance matrix generated with vegdist (vegan package v2.6–4) and the pcoa command (ape package v5.6–2) in R. Differences between HES and LES isolates were tested with a PERMANOVA test using the adonis function of the vegan package v2.6–4. Three outbreak periods were defined based on the strain incidence: early outbreak (2008–2010), late outbreak (2011–2012), and post-outbreak (2013–2018). Pairwise PERMANOVA tests were also performed for these different periods using the pairwise.adonis2 function of the pairwiseAdonis v0.4.1 package.

To highlight and rank genomic features driving the epidemic success of the ST228 MRSA clone, Random Forest (RF) models were built using the randomForest package v4.7–1.1 in R. Different models were generated from the following data sets as predictors: total genome, virulence loci, resistance loci, resistance mutations, or pathogenicity island data sets. Epidemic success categories were used as response variable in these models. Prior model training and features presenting no variation among the different strains were removed, and categorical values were transformed using one-hot encoding. The best RF model parameters were obtained for each data set by removing the less predictive features to reduce noise and optimize model prediction power, when possible. Out-of-bag (OOB) errors, precision, recall, and F1 score metrics were calculated for model performances evaluation.

## RESULTS

**Fig 1:**
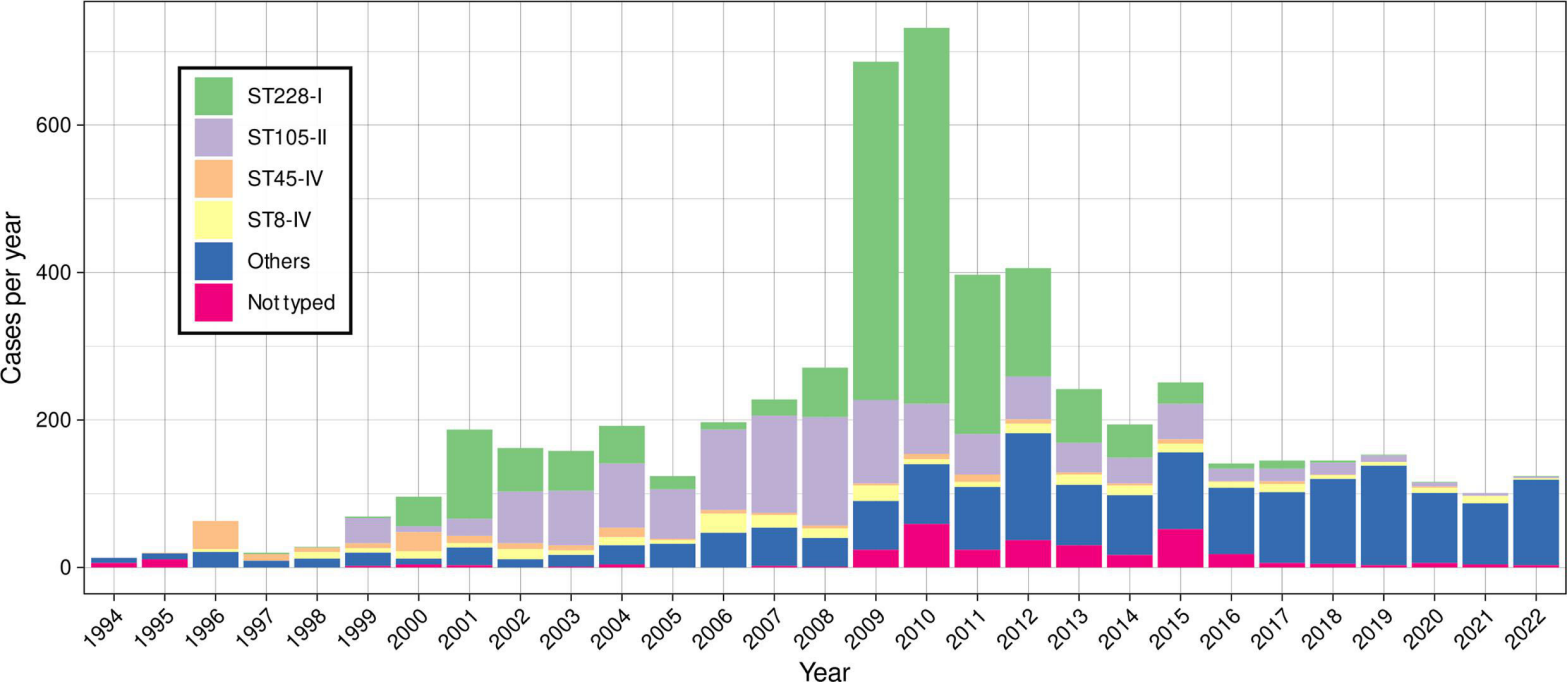
Annual incidence of major MRSA clones at the University Hospital of Lausanne from 1994 to 2022.

Since the end of the 2008–2012 outbreak, the incidence of the ST228 clone declined, and it became rare in our hospital with only a few cases yearly since 2018 (Fig. 1). Interestingly, the proportion of this ST among other STs also declined, and this clone was gradually replaced over time. From 2013 to 2018, 211 ST228 MRSA isolates were documented in our hospital. Among them, 56 were selected for further analyses. The evolutionary position of the 56 post-outbreak ST228 isolates obtained from 2013 to 2018 was determined by constructing a maximum likelihood tree with 365 sequences previously obtained from Swiss and European isolates (17) (Fig. S1). All Swiss isolates clustered within the same clade, with older isolates (1999–2003) at the root and two later sub-clusters, one including Geneva isolates (2006–2012, yellow) and the other with the Lausanne 2008–2012 outbreak (orange). The Lausanne outbreak sub-clade comprise 218 isolates recovered from 2008 to 2018.

**Fig 2:**
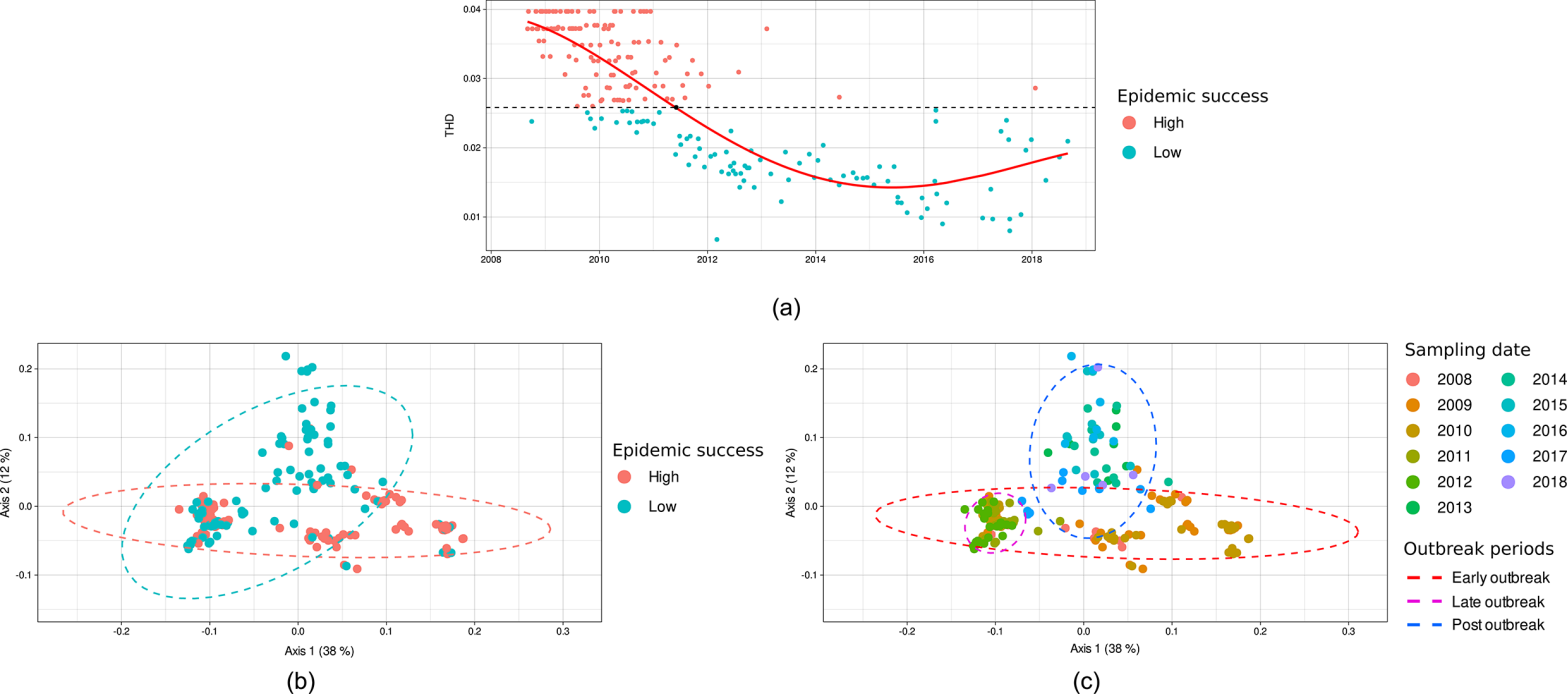
Time-scaled haplotypic density (THD) values calculated from core SNP distances using ST228 isolates from the Lausanne outbreak sub-clade (**a**). The data were fitted by a linear regression using a fourth-degree polynomial function, and the first inflection point was chosen as threshold for determining high (red) and low (blue) epidemic success isolates. PCoA of the accessory genome profiles (Jaccard distances) of the ST228 isolates from the Lausanne outbreak highlighting either the epidemicity level (**b**) or the sampling date (**c**). All clusters were proved to be significantly different (PERMANOVA, *P* < 0.004).

To obtain a metric of epidemicity, THD was calculated for determining the drivers of epidemic success in *S. aureus* population using isolates genomes from the Lausanne outbreak sub-clade as defined above (Fig. 2a). THD represents sample density for a defined timescale as represented in a phylogenetic tree. A high THD value means high density, which is related to high transmissibility and spread and therefore high epidemic success. Here, THD values showed a decline over time, with a rapid decrease between 2010 and 2012, followed by stabilization from 2012 to 2018. A linear regression model using a fourth-degree polynomial equation was the best fitting model (R^2^ = 0.69). The first inflection point was chosen as the threshold for separating HES isolates from LES isolates, splitting the data sets into 123 HES isolates and 95 LES isolates.

The core genome of the 218 isolates of the Lausanne outbreak sub-clade was composed of 2200 genes, while the accessory genome consisted of 1174 genes (Fig. S2). Among the latter, 684 (58.26 %) corresponded to genes present in a minority of isolates (0%–15%). The accessory genomes of all isolates were compared by calculating the Jaccard distances and plotted in a PCoA ordination. The two first axes represented 38% and 12% of the variance among the samples, respectively. HES and LES isolates formed two clusters that partially overlapped but were significantly different (PERMANOVA, *P* < 0.001, Fig. 2b; Table S3). The overlapping HES and LES isolates belonged to the 2011–2012 period, which corresponds to the shift between high and low THD values (Fig. 2c). Early outbreak samples (2008–2010) and post-outbreak samples (2013–2018) were clearly distinct on the ordination plot, and all clusters were significantly different (PERMANOVA, *P* < 0.001).

**Fig 3:**
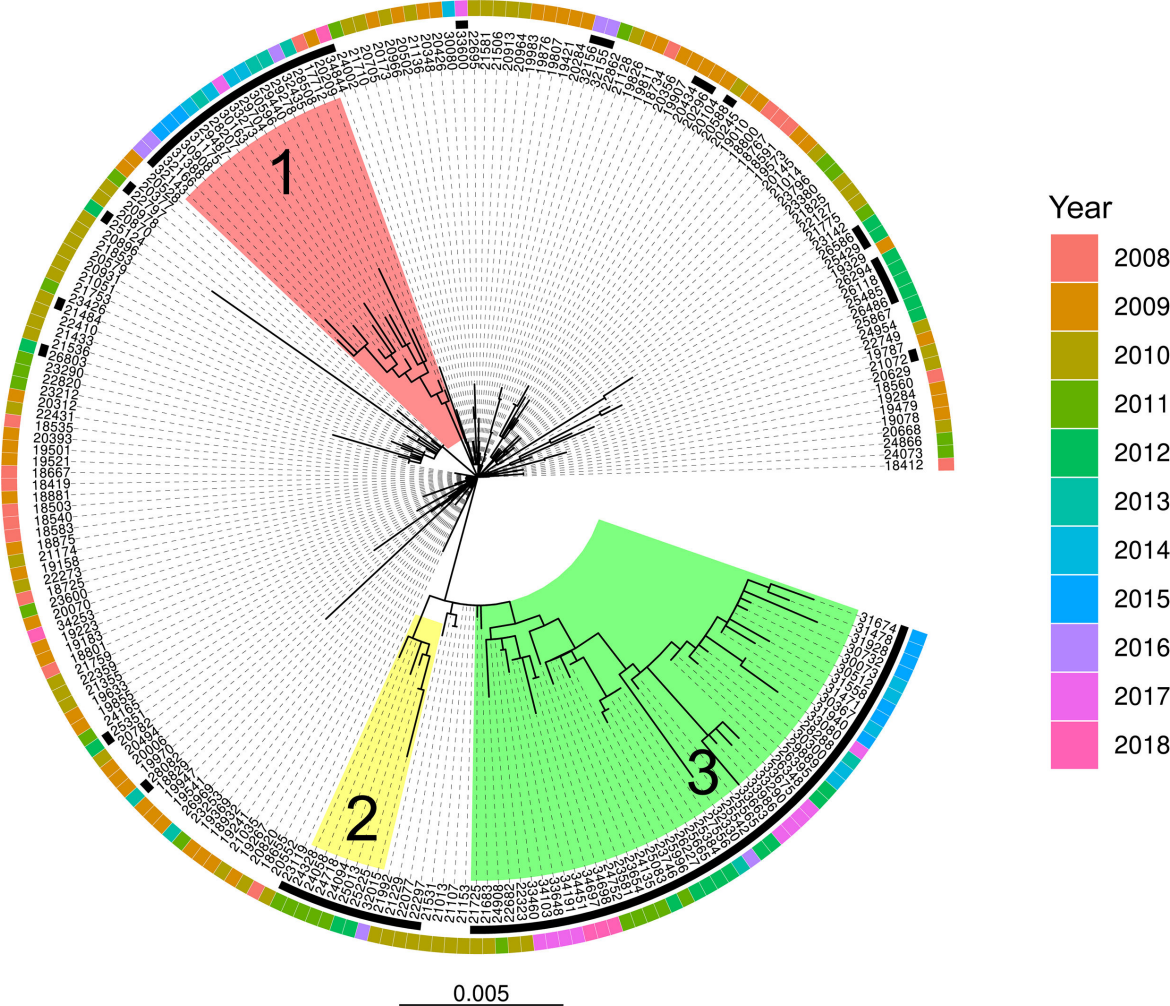
Maximum likelihood tree of the 218 ST228 isolates from the Lausanne outbreak. Low epidemic success isolates are marked with a black square. Three distinct low epidemic success clusters are highlighted in red, yellow, and green.

A maximum likelihood tree was built with all ST228 isolates from the Lausanne outbreak sub-clade (2008–2018) exclusively and rooted on the first reported case (Fig. 3). Three LES clusters were visible and represented 70 of the 95 LES isolates. The remaining 25 isolates were spread across the tree. LES cluster 1 was closely related to isolates from the early outbreak (2008–2010) and composed of isolates from 2008 to 2017. LES clusters 2 and 3 were located at the tips of the tree, representing more recently diverged lineages and composed of late outbreak isolates (2011–2012) and post-outbreak isolates (2013–2018).

**Fig 4:**
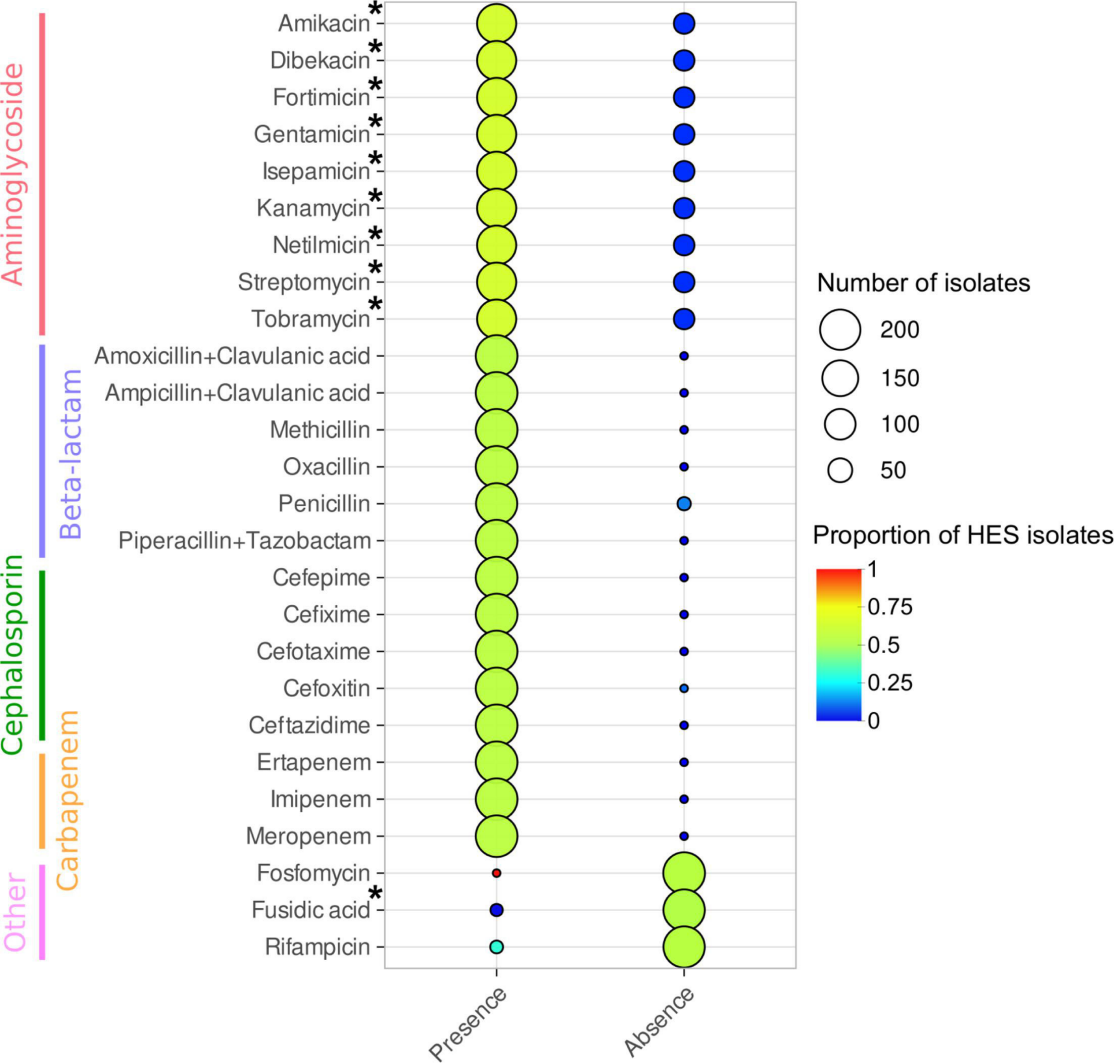
Association of predicted antibiotic resistances with epidemic success. Circle size is proportional to the number of isolates in each group, whereas circle colors represent the proportion of isolates with high epidemic success in each group. Resistance traits were marked with an asterisk when the association (Fisher’s exact test) was significant (*P* < 0.05).

Using the resistance trait data set from Bionumerics, the association between the presence of each antimicrobial resistance trait presenting variations among isolates with the epidemic success was tested (Fig. 4). The absence of resistance to aminoglycosides was significantly correlated with LES. Similarly, the absence of fusidic acid resistance was significantly associated with HES, although this trait was only present in five isolates.

**Fig 5:**
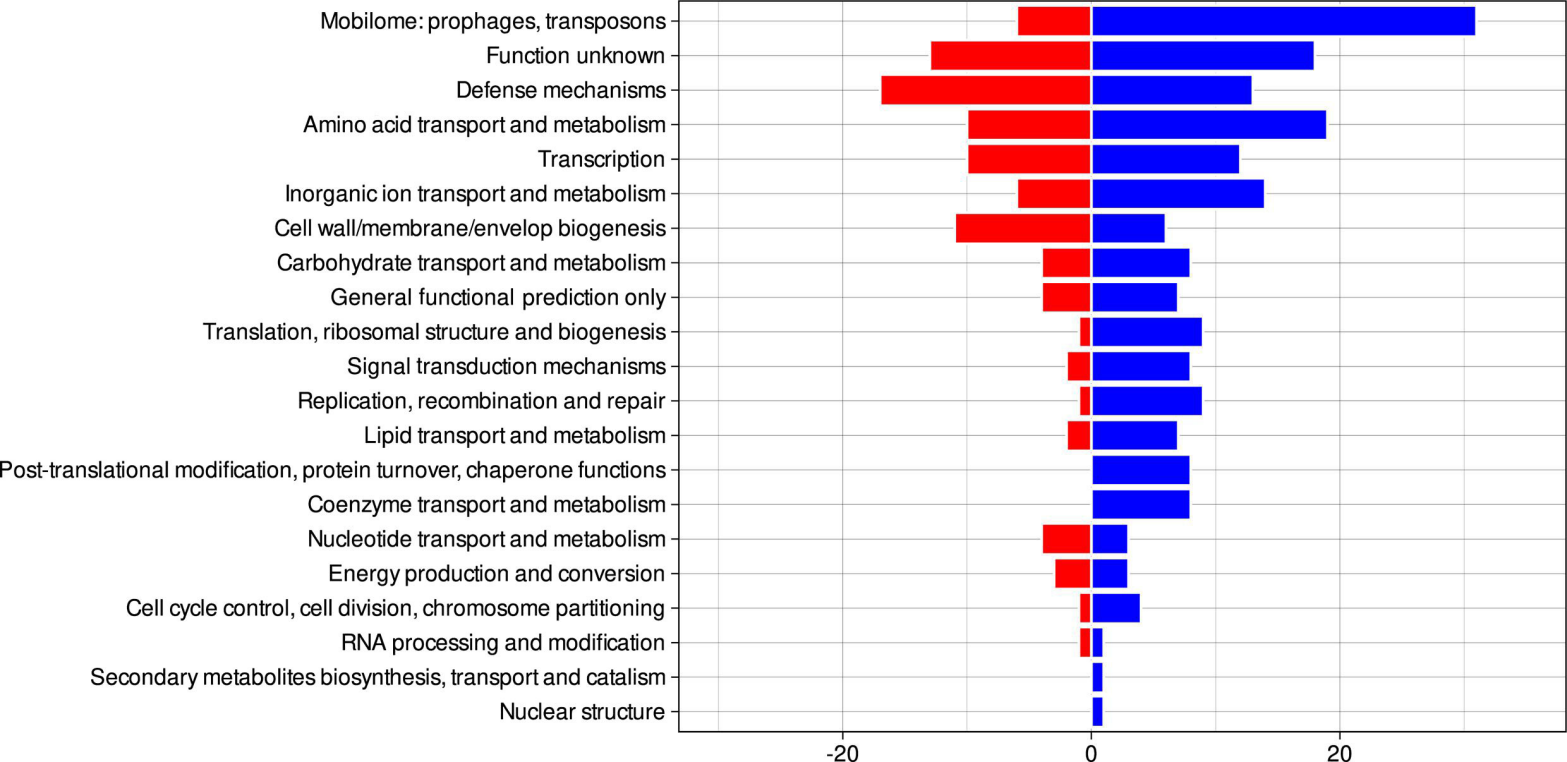
Number of genes acquired (blue) or lost (red) by low epidemic success isolates compared with high epidemic success isolates according to the Clusters of Orthologous Genes (COG) classification. Only genes with adjusted *P* < 0.05 (Benjamini–Hochberg test) and empirical *P* < 0.05 were selected from the genome-wide association study.

The GWAS showed that 264 genes were significantly associated with LES of the ST228 clone during the Lausanne outbreak. Among them, 125 appeared to be lost by LES isolates, and 139 were acquired. Most of the genes were related to the mobilome, transcription mechanisms, defense mechanisms, and amino acid transport (Fig. 5). Most mobilome genes corresponded to phage proteins, and slightly more gene acquisitions were observed compared with gene losses (Table S2). A similar number of transcription genes were acquired and lost, and they encoded for diverse transcription factors. Interestingly, most of the genes belonging to the defense mechanism category were lost (80%). This category includes mechanisms allowing the bacteria to survive to host defenses and in various environments, but it also includes virulence factors including pathogenicity island proteins, metal resistance proteins, and antiseptic resistance protein (SepA). In contrast, most genes (72.4%) relating to amino-acid synthesis were acquired in LES isolates.

These numbers of gene loss and gene gain greatly differed among the different clusters (Fig. S3). Overall, 170, 3, and 156 genes were acquired, and 92, 0, and 136 genes were lost by LES clusters 1, 2, and 3, respectively. LES cluster 2 showed barely any significant differences with HES isolates. LES clusters 1 and 3 showed a pattern of lost and acquired genes similar to that obtained when comparing all LES and HES isolates. However, only 122 genes were common to both clusters, whereas 140 and 168 genes were exclusive to LES clusters 1 and 3, respectively. Among the common genes, 21 genes related to the mobilome (mainly phage proteins) were acquired in both clusters. The ABC-type antimicrobial peptide transport systemC permease component was acquired by both LES clusters as well as toxin/antitoxin genes (*mazEF*), whereas others were lost (SSL genes, staphylococcal enterotoxin type O). Interestingly, LES cluster 1 lost toxin-associated genes *lukED* coding for a bi-component leukocidin and acquired the similar *lukGH* genes, also coding for a leukocidin. The toxin export system PMT (*pmtABC* genes) was also lost, as well as the staphylococcal enterotoxin type G. Finally, adherence genes related to virulence were sometimes lost (*eap*) and sometimes acquired (*ebp*) by LES cluster 1 isolates. In LES cluster 3, cadmium resistance genes, multidrug efflux pump QacA, and penicillin-hydrolyzing class A beta-lactamase BlaZ were lost, as well as genes related to cysteine protease staphopain A, endoribonuclease MazF, and several pathogenicity island proteins.

Several RF models were built using total genome, virulence loci, resistance loci, resistance mutations, or pathogenicity island data sets as predictors and epidemic success categories as response variables, yielding a wide range of quality metrics reported in Fig. S4; Table S4. The model using the total genome data set showed the best performance among all models with the lowest OOB error (12.39%) and the best F1 score (0.89, Fig. S4). Models using other data sets performed poorly with low recall values and high OOB error values. To highlight the most influential genes, the best predictors of the most performant model were extracted. Most of the best predictors were poorly identified or labeled with an unknown function (Table 1, complete list in Table S5). The best predictor was identified as a carboxypeptidase regulatory-like domain-containing protein. This domain is present in sequences that are variously annotated, such as hypothetical/conserved/membrane/cell surface protein or side tail fibre protein homolog from lambdoid prophage Rac. Moreover, almost half of the members belonging to this orthologous group were identified as fibrinogen-binding proteins, suggesting that this gene could potentially enhance isolate adhesions. This gene was characteristic of LES isolates and significantly acquired by LES clusters 1 and 3 (Table S2). The Ser-Asp rich fibrinogen-binding bone sialoprotein-binding protein *sdrE* was also a very good predictor but did not appear to be significantly associated with LES isolates. This gene is involved in bacterial cell surviving against the human immune system by binding complement factor H as an immune-evasion tactic (29). The hyperosmolarity resistance protein Ebh was also a good predictor and related to increased pathogenicity in staphylococcal infections (30). Although this gene was significantly lost by LES cluster 1, it was significantly acquired by LES cluster 3. Finally, the AraC family transcriptional regulator Rsp was a good predictor in the classification model and significantly lost by LES clusters 1 and 3. This gene encodes for a transcriptional regulator of virulence factors in *S. aureus* (31).

**TABLE 1:** Best 20 predictors of the Random Forest classification models using all genome data as predictors, sorted by mean decrease accuracy^*a*^

Group name	Description	Mean decrease accuracy
Carboxypeptidase regulatory-like domain-containing protein	Carboxypeptidase regulatory like domain containing protein / fibrinogen-binding proteins	17.56
Group_2986	Hypothetical protein	9.35
Hyperosmolarity resistance protein Ebh	Hyperosmolarity resistance protein Ebh	8.32
Group_1351	DUF1433 domain-containing protein	7.93
Group_1350	DUF1433 domain-containing protein	7.58
RxLR effector protein	RxLR effector protein	6.40
ykfC	Retron St85 family RNA-directed DNA polymerase	6.31
Cell wall-associated fibronectin-binding protein	Cell wall associated fibronectin-binding protein	6.05
Group_1091	Transposase	6.02
rsp	AraC family transcriptional regulator Rsp	5.79
sdrE	Ser-Asp rich fibrinogen-binding bone sialoprotein-binding protein	5.66
Group_539	Hypothetical protein	5.62
Group_521	Uncharacterized membrane protein YkvI/branched-chain amino acid transport system II carrier protein	5.44
Group_700	YebC/PmpR family DNA-binding transcriptional regulator	5.42
yzzA	General stress protein 26 (function unknown)	5.17
pdxS	Pyridoxal 5'-phosphate synthase subunit PdxS	5.16
Uncharacterized protein SAV2481	Uncharacterized protein SAV2481	5.15
Group_1466	Nucleoside permease NupC	4.94
Group_285	Ribosomal protein S18 acetylase RimI and related acetyltransferases	4.84
dppD	ABC-type dipeptide/oligopeptide/nickel transport system C ATPase component	4.82

^*a*^ The names of the orthologous groups were attributed by Roary. Multiple descriptions are reported when a high proportion of members of the group had a different annotation.

## DISCUSSION

In this study, we investigated the genomic changes of an epidemic clone up to 10 years after a significant outbreak. This is the first study to use whole-genome sequencing to explore the genomic evolution of post-outbreak MRSA isolates of an epidemic clone. Our results showed that all post-outbreak isolates were descendants of the recent lineage formed by the Lausanne and Geneva epidemic clones recovered from 2006 to 2012. Interestingly, the ST228 clone was less present in the second structured European survey of MRSA (32) than in the first survey (33), indicating a general decline of this clone in all countries where it was present. Although the expansion of the different MRSA lineages observed worldwide is strongly associated with key genetic acquisitions (34, 35), the reasons why MRSA declined in many countries remains poorly understood (36). The analysis of the post-outbreak isolates in the Lausanne cluster confirmed that these isolates were all descendants of the original lineage and not new introductions via patient transfers. Using THD as a metric of the clone epidemicity, we observed that the THD values decreased over the years to stabilize at a plateau around 2014–2015. Between 2010 and 2012, a higher number of LES isolates were progressively observed, showing a transition in the clone epidemicity. This correlated with the decrease of the clone incidence and the introduction of stronger infection control measures, which likely contributed mainly to the decline of the clone in our hospital. Most of the LES isolates formed three divergent sub-lineages, indicating that subpopulations emerged following the decrease of the epidemicity.

The plasticity of the bacterial genome is a key feature of bacteria adaptative capacities (37). Their genomes are in constant mutation, modeled by acquisition and loss of genetic elements that influence their fitness. The accessory genome is thought to be the main driver of *S. aureus* pathogenicity, with MGEs playing an important role in MRSA genomic evolution and adaptation (38), as confirmed for the ST228 clone (16, 17). The diversity of the accessory genome of the clone evolved over time, with clear transition from an early outbreak period to a late outbreak period and finally to a post-outbreak period. These transition periods corresponded to the transition from HES to LES isolates. The post-outbreak isolates significantly differed from the early and late outbreak isolates. Moreover, this cluster presented a higher diversification probably linked to the emergence of divergent sub-lineages as well as the extended period over which the LES isolates spread.

LES isolates were characterized by the loss of predicted resistance to antibiotics belonging to the aminoglycoside class and to fusidic acid. In their study of different clonal complexes from various countries across Europe, Baede et al. also showed that gentamicin was negatively correlated with THD (21). However, their attempt to correlate the epidemic success of the different clonal complexes with specific mutations did not reveal any marker of success. In this study, many genes were significantly associated with the epidemicity of the ST228 clone, underlining the evolution of its genome during the years following the end of the outbreak. In our case, phages appeared to be the main vector of evolutionary changes since LES isolates acquired and lost a high number of mobilome (and phage) related genes. Phages are known to be the primary factor driving the evolution of *S. aureus* genome, contributing to the emergence of successful virulent lineages. Indeed, *S. aureus* is not considered to be naturally competent, but many virulence factors leading to strains success are carried by phages (39). Although the decrease of epidemicity was probably due to the implementation of new infection control measures, it was also correlated with the loss of several genes related to virulence (toxins, adherence) or resistance, suggesting a decreased virulence of the clone. The different clusters formed by the emergent sub-lineages of LES isolates showed a similar genomic profile, but some of the genes acquired or lost were exclusive to each cluster. This correlates with the fact that they emerged at different times and suggests that these sub-lineages interacted with different phages. Acquisition and loss events in the bacterial genome can be influenced by various factors. In this case, the selection pressure encountered by the isolate in a novel environment, such as changes in hospital settings and reinforced infection control measures, may have driven these events.

As a multivariate analysis, RF models allowed us to address the relationship between epidemicity and the genetic characteristics of the clone. RF models have been used extensively and successfully with genomic profiles of various bacterial species, including *S. aureus*, to link the presence of genes with different factors, such as the susceptibility to antibiotics, hypervirulence isolate prediction, or identified promoter sequences (40–42). The epidemicity of the clone could not be attributed to a single factor, such as virulence, resistance, mutations, or pathogenicity islands. This was demonstrated by the low performance of the models using focused data sets based on these individual factors. Instead, the good performance of the models using the total genome as predictors confirmed that the epidemicity of the clone was more likely due to a combination of multiple factors. The most important predictor of the epidemicity was represented by a gene potentially related to fibrinogen-binding. This gene was essentially present in LES isolates and thus represents a marker of some post-outbreak isolates. GWAS showed that this gene was acquired by LES cluster 2 and 3 isolates. Two other proteins, a cell wall-associated fibronectin-binding protein and a Ser-Asp-rich fibrinogen-binding bone sialoprotein-binding protein, appeared among the best predictors of LES. Fibrinogen-binding and fibronectin-binding proteins are essential for the attachment of *S. aureus* to the host cells (43, 44). It is thought to highly contribute to *S. aureus* virulence by allowing the bacteria to form clumps in the fibrin layer and to hide from the host immune system and from antibiotics. By contrast, the loss of many genes related to defense mechanisms, encompassing strategies for evading host defenses and toxin production, implies a reduced virulence potential of the LES isolates. In *S. aureus*, genes associated with virulence, particularly those found within pathogenicity islands and those encoding toxins, are typically harbored by phages (38). Furthermore, numerous transcriptional genes have been either gained or lost in LES isolates, some of them known for playing a role in the virulence of *S. aureus*. Notably, the AraC family transcriptional regulator Rsp and various AraC-type DNA-binding domains were depleted, whereas DNA-binding LacI/PurR family transcriptional regulators were acquired (45, 46). Possible alterations in the regulation of virulence within the LES isolates may contribute to the loss of epidemicity alongside the modifications of virulence factors. The acquisition of attachment factors and the reduced virulence of the LES isolates imply that these isolates may possess characteristics enabling them to persist inconspicuously within the host. Of note, Senn et al. characterized the ST228-I clone as a stealthy superbug, prevalent among numerous long-term asymptomatic carriers (16). Furthermore, the ST228-I clone has been described as adapted to enteric carriage, which might have also influenced its evolutionary course. In addition to the factors previously mentioned, many other genes emerged as good predictors. These findings underscore the multifactorial nature of the drivers behind the evolution of this clone.

It is challenging to determine the precise reasons of the ST228 clone decline, and multiple factors may have contributed (7, 47, 48). The progressive replacement of clones was unlikely here, as the total incidence of MRSA declined, and the ST228 clone was not replaced by another predominant clone. Another explanation could be the accumulation of SNVs and the genetic drift of the clone, decreasing its fitness (49). The success of the new infection control policy and antibiotic stewardship remains the most likely possibility and is reflected by the overall decrease of all STs. However, it hardly explains the decrease in the proportion of ST228 amongst the other STs.

Studies, such as this one, are subject to several limitations that may impact the outcomes and conclusions. First, when investigating the biological factors underlying the decline of a clone, it is difficult to observe the true unsuccessful isolates/lineages that lacked these determinants, as they may have become extinct. Additionally, investigations of outbreak dynamics inherently result in unbalanced data sets, with most isolates representing the outbreak period and relatively few isolates collected during the post-outbreak period. Finally, *S. aureus* annotation challenging due to inconsistencies in annotated genomes present in the databases (50). To date, no curated database of *S. aureus* genome exists, comparable to the Pseudomonas Genome Database for *Pseudomonas* species (51).

Numerous studies have investigated the genetic factors underlying the emergence of MRSA lineages. Understanding the characteristics of successful lineages is crucial for improving transmission control. However, only a limited number of studies have examined the genomic basis for the decline of epidemic clones. As far as we are aware, this is the first study comparing the genomic content of isolates from the outbreak period with those collected up to 10 years later for the same clone. We showed the emergence of sub-lineages after the outbreak period, all of which continued to circulate within the hospital until the clone nearly vanished. Through both univariate and multivariate analyses, we illustrated that the diminishing epidemiological success of the clone was linked to the acquisition and loss of numerous genes, presumably as a result of interactions with phages. These alterations may have potentially driven the clone towards greater adaptability for long-term carriage while simultaneously reducing its virulence, but they did not allow the clone to persist in the population since it almost completely disappeared.

## Data Availability

All reads generated for this study were deposited in the EMBL Nucleotide Sequence Data Base (ENA) with the project number PRJEB49254.

## SUPPLEMENTAL MATERIAL

Fig. S1 jcm.00203-24-s0001.eps Maximum likelihood tree of the 421 ST228 strains from Lausanne, Geneva, and other European hospitals.

Fig. S2 jcm.00203-24-s0002.eps Pan-genome composition of the Lausanne outbreak isolates.

Fig. S3 jcm.00203-24-s0003.eps Number of acquired or lost genes by the isolates from the 3 low-epidemic-success clusters to isolates with high epidemic success.

Fig. S4 jcm.00203-24-s0004.eps Quality metrics of the Random Forest models using the different genomics datasets as predictors and epidemic success categories as response variable.

Supplemental legends jcm.00203-24-s0005.docx Legends for Fig. S1 to S4.

Supplemental tables jcm.00203-24-s0006.xlsx Tables S1 to S5.

ASM does not own the copyrights to Supplemental Material that may be linked to, or accessed through, an article. The authors have granted ASM a non-exclusive, world-wide license to publish the Supplemental Material files. Please contact the corresponding author directly for reuse.
