# Supplementary material for: Genomic evolution of ST228 SCCmec-I MRSA 10 years after a major nosocomial outbreak
Source: J Clin Microbiol. 2024 Jun 27;62(7):e00203-24. doi: 10.1128/jcm.00203-24 (PMC11250417; doi:10.1128/jcm.00203-24)
Supplement: Supplemental legends — Legends for Fig. S1 to S4. [file jcm.00203-24-s0005.docx]

Supplementary figures captions

Figure S1: Maximum likelihood tree of the 421 ST228 strains from Lausanne, Geneva and other European hospitals. The yellow area includes mostly strains from Geneva after 2006. Orange area includes almost all strains of the Lausanne outbreak. All Swiss isolates clustered within the same clade, with older isolates (1999-2003) at the root and two later sub-clusters, one including 2006-2012 Geneva isolates (yellow sub-clade) and the other one composed of Lausanne 2008-2012 outbreak, Vaud isolates and post-outbreak isolates (orange sub-clade).

Figure S2: Pan-genome composition of the Lausanne outbreak isolates.

Figure S3: Number of acquired (blue) or lost (red) genes by the isolates from the 3 low epidemic success clusters to isolates with high epidemic success. The genes were classified according to the Clusters of Orthologous Genes (COG)classification. Only genes with an adjusted p-value < 0.05 (Benjamini–Hochberg test) and an empirical p-value < 0.05 were selected from the Genome Wide Association Studyanalysis.

Figure S4: Quality metrics of the Random Forest models using the different genomics datasets as predictors and epidemic success categories as response variable.
